# Expression and Molecular Evolution of Two *DREB1* Genes in Black Poplar (*Populus nigra*)

**DOI:** 10.1371/journal.pone.0098334

**Published:** 2014-06-02

**Authors:** Yanguang Chu, Qinjun Huang, Bingyu Zhang, Changjun Ding, Xiaohua Su

**Affiliations:** State Key Laboratory of Tree Genetics and Breeding, Research Institute of Forestry, Chinese Academy of Forestry, Key Laboratory of Tree Breeding and Cultivation, State Forestry Administration, Beijing, China; National Institute of Plant Genome Research, India

## Abstract

Environmental stresses such as low temperature, drought, and high salinity significantly affect plant growth and yield. As selective forces, these adverse factors play essential roles in shaping phenotypic variation in plant populations. Black poplar (*Populus nigra*) is an economically and ecologically important forest tree species with widely distributed populations and is thus suitable for experiments detecting evolutionary footprints left by stress. Here, we performed expression and evolutionary analysis of two duplicated DREB A1-subgroup (DREB1) genes, *PnDREB68* and *PnDREB69*, encoding transcription factors that are involved in stress responses. The two genes showed partially overlapping but distinct expression patterns in response to stresses. These genes were strongly and rapidly induced by cold stress in leaves, stems, and roots. In leaf tissue, dehydration stress induced the expression of *PnDREB68* but not *PnDREB69*. *PnDREB69* displayed more rapid responses and longer expression durations than *PnDREB68* under salt and ABA stress, respectively. Based on single nucleotide polymorphism (SNP) analysis, we found significant population genetic differentiation, with a greater *F*_ST_ value (0.09189) for *PnDREB69* than for *PnDREB68* (0.07743). Nucleotide diversity analysis revealed a two-fold higher π_T_ for *PnDREB68* than for *PnDREB69* (0.00563 vs. 0.00243), reflecting strong purifying selection acting on the former. The results suggest that positive selection acted on *PnDREB69*, as evidenced by neutral testing using Tajima’s *D* statistic. The distinct selective forces to which each of the genes was subjected may be associated with expression divergence. Linkage disequilibrium (LD) was low for the sequenced region, with a higher level for *PnDREB68* than for *PnDREB69*. Additionally, analysis of the relationship among carbon isotope ratios, SNP classes and gene expression, together with motif and domain analysis, suggested that 14 polymorphisms within the two genes may be candidates for an association study of important traits such as water use efficiency/drought tolerance in black poplar.

## Introduction

The growth and development of plants are frequently challenged by environmental stresses such as drought, high salinity, and temperature change [1], resulting in a significant reduction in productivity in both crops and forest trees. These adverse factors represent major selective forces that act on plant phenotypes [2]. Plant species with widely distributed natural populations constantly exhibit phenotypic differentiation in many traits due to adaptation to their specific environments. This intraspecific variation is thought to be largely shaped by the forces of natural selection [3], [4]. Thus, understanding the genetic basis underlying adaptive phenotypic variation is an important aim for both evolutionary genetics and molecular breeding in plants [5].

Stress tolerance is a quantitative trait that is often modulated by a group of transcription factor genes. CBF/DREB (C-repeat binding factor/dehydration-responsive element binding) is a gene family belonging to the AP2/ERF (APETALA2/EREBP) superfamily in plants, which encodes a large number of transcription factors [6]. CBF/DREB genes specifically bind to the C-repeat (CRT)/dehydration-responsive element (DRE) motif and regulate the expression of various stress-responsive genes that harbor these elements [7]. The DREB subfamily is composed of six subgroups of genes (A1–A6) [8]. Among these genes, in addition to containing a highly conserved AP2/ERF DNA-binding domain that is ubiquitous to all DREB genes, most A1-subgroup (DREB1) proteins possess conserved sequences including the nuclear localization signal (NLS) sequence PKRAGRTKFRETRHP, the DSAW motif, and the LWSY motif [9]. DREB1 genes, namely *CBF1*–*CBF4*, *DDF1*, and *DDF2*, were first identified in the model plant *Arabidopsis thaliana* [10]–[13]. Genes belonging to the DREB1 subgroup were subsequently identified and functionally characterized in many annual herbaceous plants, such as rice (*Oryza sativa*), wheat (*Triticum aestivum*), barley (*Hordeum vulgare*), maize (*Zea mays*), and *Atriplex hortensis* [7]. Regarding perennial woody plants, studies of *DREB1* genes have been only documented in a limited number of species, including *Populus* spp [14], [15], *Eucalyptus* spp [16], [17], and peach (*Prunus persica*) [18], [19].

The majority of studies of DREB genes have focused on the molecular functions of individual gene; an understanding of the mechanisms of functional divergence among DREB genes, and studies aimed at detecting the evolutionary footprints on these genes, are currently lacking. A previous study has proposed that the functional divergence of three duplicated members of the DREB1 gene family (CBF1, CBF2, and CBF3) may have resulted from specific sequence polymorphisms that were produced by contrasting evolutionary forces in *Arabidopsis* [20]. However, to date, the evolutionary history and molecular genetic analysis of the functional divergence of *DREB1* genes have not been reported for any long-lived woody tree species.

Poplars are employed as a model woody species in plant biology studies, particularly studies involving adaptation, secondary growth, and the natural variation of traits [21]. *Populus nigra* L. (black poplar) belongs to section *Aigeiros* within the genus *Populus* (family Salicaceae) [22]. *P. nigra* is predominantly outbreeding, with a wide distribution across Europe, the Mediterranean coast of Northern Africa, and Western and Central Asia [23]. *P. nigra* has long been used to obtain interspecific hybrids (e.g., *Populus*. × *euramericana*) with favorable traits for commercialized planting by crossing this plant with related species, such as *Populus deltoides*. Natural populations of *P. nigra* also play important roles in the balance of riparian ecosystems by restoring riverbanks and controlling flooding [24]. Populations of *P. nigra* are distributed across a wide range of areas, from arid regions in Italy to relatively humid regions in Germany. This ecological feature makes *P. nigra* a suitable species to investigate the genetic factors that contribute to the natural variation of adaptation-related traits in plants such as drought tolerance. In this study, we combined expression analysis and molecular evolution estimation, including nucleotide diversity, neutrality testing, and linkage disequilibrium, using polymorphism data from two duplicated *DREB1* genes, *PnDREB68* and *PnDREB69*, to infer their diverged expression patterns in response to stresses and the selection forces that may have contributed to this divergence. We also examine the potential implications of our results for association studies of drought tolerance/water use efficiency traits.

## Materials and Methods

### Ethics Statement

No specific permissions were required for the described field studies. The Jade Spring Hill Nursery Garden in Beijing is not privately-owned.

### Plant Materials

One-year-old *P. nigra* (clone ‘N21’) trees were grown in a greenhouse under controlled conditions at the Chinese Academy of Forestry (CAF) for gene expression analysis. To examine molecular evolution based on single nucleotide polymorphisms and phenotypic analysis, a total of 65 unrelated individuals representing the major distribution of *P. nigra* were planted in a field trial at the Jade Spring Hill Nursery Garden in Beijing. These individuals were initially sourced from natural populations across Europe and Asia, including four geographic regions (WE, Western Europe; CE, Central Europe; SE, Southern Europe; and CA, Central Asia) that cover 15 countries. Detailed information about each sample tree is presented in the Table S1. Young, healthy leaf tissues were sampled from the 65 individuals, quickly frozen in liquid nitrogen, and stored at −70°C until use.

### Cloning of cDNAs and Amplification of their Genomic DNA

A genome-wide analysis of the AP2/ERF superfamily of poplar (*Populus trichocarpa*) revealed six gene members of the DREB1 subgroup [25]. Within this subgroup, two *DREB1* genes, *DREB68* (XM_002299529) and *DREB69* (XM_002298031), are located on chromosome 1 and appear to be tandem duplicates arranged in a tail-to-tail manner. The two genes were not involved in the whole-genome duplication (WGD) event and the intergenic region between them is relatively short; thus, they are suitable for the analysis of expression divergence and molecular evolution. The counterparts in *P. nigra* were therefore named *PnDREB68* and *PnDREB69*. Total RNA was extracted from leaf tissue using an Ambion Plant RNA Isolation Aid (Life Technologies, Carlsbad, CA, USA) according to the manufacturer’s instructions. Gene-specific primers were designed for cDNA cloning via RT-PCR based on the sequences of *DREB68* and *DREB69* transcripts and the publicly available sequences of the two loci from JGI (http://genome.jgi-psf.org/Poptr1_1/Poptr1_1.home.html; http://www.phytozome.net/search.php?method=Org_Ptrichocarpa) [26]. Genomic DNA for each individual tree was extracted from leaf tissue using a DNeasy Plant Mini Kit (Qiagen, Hilden, Germany) following the manufacturer’s instructions. Primer pairs for amplifying genomic DNA in *P. nigra* were designed according to the genome assembly for *P. trichocarpa* described above. The web-based program Primer 3 [27] was used to design primers for RT-PCR and genomic DNA amplifications. Expected PCR products were purified and subcloned into the pGEM-T vector (Promega, Fitchburg, WI, USA). To amplify genomic DNA, reactions were carried out in a volume of 50 µl containing 50 ng of template DNA, 0.2 mM of each dNTP, 0.5 µM of each primer, 1× PCR buffer plus MgCl_2_, and 0.5 U LA-Taq polymerase (TaKaRa, Dalian, China). All PCR reactions were run on a PE 9700 Thermal Cycler (Applied Biosystems, Foster City, CA, USA). Detailed information about the primers and PCR programs can be found in Table S2. Using a BigDye Terminator 3.1 Kit (Applied Biosystems), recombinant plasmids from RT-PCR were sequenced, and PCR products produced from genomic DNA templates were purified and subjected to direct sequencing. Sequencing reactions were conducted using an ABI 3730XL Automated DNA Sequencer (Applied Biosystems). Each amplicon was sequenced on both strands to generate a consensus sequence and to minimize PCR and sequencing errors.

*Cis*-acting elements within the predicted promoter sequences were deduced by searches in the PlantCARE and PLACE databases [28]–[30]. The deduced amino acid sequences of *PnDREB68* and *PnDREB69* were aligned using Clustal X [31] to perform a comparison analysis of gene structure.

### Stress Treatments, qRT-PCR, and Microarray Analysis

Cuttings of *P. nigra* were cultivated in plastic pots containing an experiment-specific soil mixture in a greenhouse. When the plants were approximately 20 cm tall, they were exposed to cultivation solution containing 300 mM NaCl, 20% polyethylene glycol (PEG6000), or 100 mM abscisic acid (ABA) and grown at a low temperature (4°C) for 0 (normal growth conditions without stress), 2, 8, 24, or 48 h. Each treatment consisted of five replicates. Following the treatments, the leaves, stems, and roots were collected, frozen immediately in liquid nitrogen, and stored until use. Total RNA was extracted from the collected tissues using an Ambion Plant RNA Isolation Aid (Applied Biosystems) according to the manufacturer’s instructions. The cDNA was synthesized using a PrimeScript RT Reagent Kit (TaKaRa). Then, qRT-PCR was performed in an ABI 7500 FAST Sequence Detector (Applied Biosystems) with SYBR Green Real-time PCR Master Mix (TaKaRa). Gene-specific primers were designed to amplify 120–130 bp fragments of *PnDREB68* and *PnDREB69*, and parallel PCRs were carried out using a gene-specific primer pair for poplar *ACTIN1* (GenBank Accession XM_002298674) as a reference gene. Primer sequences for the real-time PCR assay of *PnDREB68*, *PnDREB69*, and *ACTIN1* are listed in Table S2. Four technical replicates for each PCR reaction were performed per RNA sample.

To perform comparisons of the orthologs within the genus *Populus*, expression profiles of *DREB68* and *DREB69* from *P. trichocarpa* in a range of samples were analyzed using the publicly available Affymetrix microarray data for poplar from the PopGenExpression server (http://www.barutoronto.ca) [32]. The dataset can also be downloaded from the Gene Expression Omnibus (http://ncbi.nlm.hih.gov/geo) under the accession number GSE13990. Probe sets for *P. trichocarpa DREB68* and *DREB69* gene models were retrieved using the Probe Match tool in the NetAffx Analysis Center (http://www.affymetrix.com/analysis/index.affx).

### Phylogenetic Analysis

To infer the general evolutionary history of plant *DREB1* genes, amino acid sequences of representative members of the A1 group of DREB family (DREB1) proteins from five species (*P*. *trichocarpa* [25], *Arabidopsis thaliana*, *Oryza Sativa*, *Sorghum bicolor*, and *Vitis vinifera*) were randomly selected and retrieved from the whole-genome databases in Phytozome v9.1 at www.phytozome.net (Dataset S1). Thus, the sequences included in this analysis did not specifically consider genes created by WGD or tandem duplication. These sequences, along with those of *PnDREB68* and *PnDREB69*, were subjected to phylogenetic analysis using MEGA 5 [33]. Sequences from members of the A2 group of DREB family (DREB2) proteins in moss (*Physcomitrella patens*) were also downloaded from Phytozome and used as an outgroup (Dataset S1). The neighbor-joining method was used to build phylogenetic trees with the p-distance amino acid substitution model. Phylogeny testing was performed using 1,000 bootstrap replicates.

### Nucleotide Diversity and Population Differentiation

Genomic DNA regions containing *PnDREB68* and *PnDREB69* were sequenced in 65 unrelated individuals. Sets of alleles for each locus were aligned using Clustal W [31]. Single nucleotide polymorphisms (SNPs) were identified and counted by visually inspecting each sequence alignment for each locus. The corresponding chromatogram files were checked to confirm each polymorphic site and its genotype class. Nucleotide diversity was estimated using DnaSP v5.10 [34] (http://www.ub.es/dnasp/). The levels of nucleotide diversity of the candidate genes were estimated as θw and π, which are based on the number of segregating sites [35] and the average number of nucleotide differences per site between sequences [36], respectively. Insertions and deletions (indels) were excluded from these analyses. Population genetic differentiation was estimated as the fixation index, *F*_ST_ [37], [38] between pairs of populations (geographic regions in this study) using DnaSP. The significance of population differentiation was evaluated using the *S*_nn_-test statistic of Hudson [39].

### Tests of Neutrality

To test for departures from the standard Wright-Fisher model of neutral evolution, statistics including Tajima’s *D* [40] and Fu and Li’s *D* [41] were calculated for both whole-gene and coding regions, synonymous (syn) sites, nonsynonymous (nonsyn) sites, and silent (synonymous and noncoding) sites using DnaSP. Fu and Li’s *D* is believed to be more sensitive than Tajima’s *D* for detecting evolutionary events such as population expansion and positive selection [41]. Neutral testing was performed using SNP data obtained from each geographic region or whole population. The significance of these statistics was tested by 1,000 replicates of coalescent simulation based on Pi [42].

### Linkage Disequilibrium

Pairwise linkage disequilibrium across the *PnDREB68* and *PnDREB69* loci was estimated as the squared correlation of allele frequencies, *r* ^2^ [43]. The value between pairs of SNPs (minor allele frequency >0.10) was measured using TASSEL software [44], with one-tailed Fisher’s exact tests and Bonferroni corrections applied for each locus. The statistical significance of each value was determined using Fisher’s exact tests.

### Measurement of Carbon Isotope Ratio

Carbon isotope ratio (δ^13^C, ‰), or carbon isotope composition, was proposed as an indicator of water use efficiency, which can reflect drought tolerance in plants [45]. Leaf tissues were collected from the 65 clones in September. This time point was chosen because during September, bud set was observed in all trees, which indicates the end of the growing season. The leaves were dried in an oven at 70°C and ground to a fine powder. The samples then were sent to the MASS Laboratory of Institute of Atom at the Chinese Academy of Agricultural Sciences, and the carbon isotope ratio of each sample (^13^C/^12^C_sample_) was measured using a MAT-251 Isotope Ratio Mass Spectrometer (Finnigan, Waltham, MA, USA). These ratios were expressed relative to the V-PDB standard (R_PDB_), where δ^13^C (‰) = (^13^C/^12^C_sample_ − ^13^C/^12^C_PDB_)/(^13^C/^12^C_PDB_)×1,000. Carbon isotope discrimination values were not shown, as the carbon signature of the trial field was not identified. For each treatment, the ninth leaf from each of three trees was collected and mixed for subsequent analysis. Thus, a total of nine leaves per individual were sampled and three replicated values were thereby obtained. Differences in carbon isotope ratios among 65 individuals, as well as among individuals for each SNP, were evaluated by one-way analysis of variance (ANOVA, α = 0.05). Statistical analyses were performed using Data Processing System (DPS) 3.01 software [46].

## Results

### Characterization of cDNA and Genomic DNA Sequences at the *PnDREB68* and *PnDREB69* Loci

In *P. trichocarpa*, the two *DREB1* genes examined are annotated in NCBI as *DREB68* (XM_002299529) and *DREB69* (XM_002298031), and the corresponding gene models in the genome database are annotated as Potri.001G110800 and Potri.001G110700, respectively. Using primers designed with these sequences, the cDNAs with complete coding sequences (CDSs) for the two genes were isolated from *P. nigra* using RT-PCR (GenBank accession numbers KJ000090 and KJ000091). These two genes were designated as *PnDREB68* and *PnDREB69* according to the nomenclature for the *P. trichocarpa* orthologs in NCBI. *PnDREB68* and *PnDREB69* have 753-bp and 693-bp open reading frames encoding polypeptides of 251 and 231 amino acid residues, respectively. We also isolated 111 bp and 93 bp of the 5′UTR and 183 bp and 234 bp of the 3′UTR for *PnDREB68* and *PnDREB69*, respectively. These two genes share 57.32% nucleotide sequence and 54.75% amino acid sequence identities. A comparison of *PnDREB68* and *PnDREB69* with homologs in *P. trichocarpa* indicated homologous DNA sequence identities of 60.94% and 97.09%, and amino acid sequence homologies of 77.99% and 98.27%, against *DREB68* and *DREB69*, respectively.

**Figure 1:**
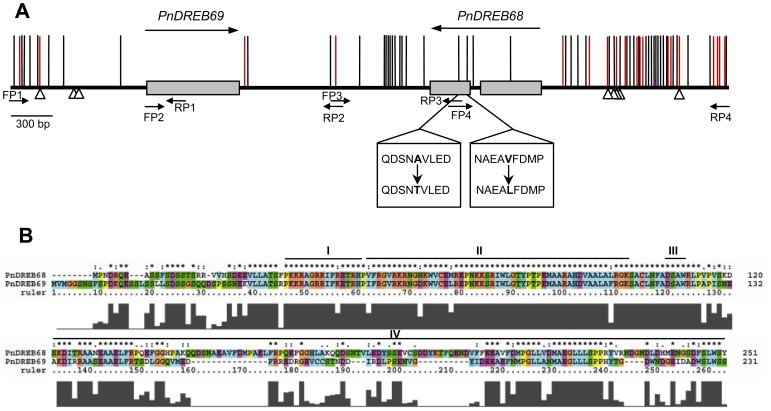
Genomic organization, polymorphism distribution (A), and comparison of protein sequences (B) of *PnDREB69* and *PnDREB68*. (A) Noncoding regions, including the promoter, intron, and intergenic sequence are represented by lines, and coding regions are indicated by boxes. The arrows represent the orientation of transcription (above the gene structure) or the locations of PCR primers (below the gene structure). The vertical lines above the gene structure indicate common SNPs (frequency >0.10) (black) and indels (red). Triangles below the gene structure indicate *cis*-acting elements with changed sequences caused by SNPs or indels. Two nonsynonymous SNPs that caused changed amino acid codons are illustrated below the coding regions. (B) Alignment of PnDREB69 and PnDREB68 proteins. Nuclear localization signal (NLS, I), AP2 domain (II), DSAW motif (III), and putative activation domain are indicated by lines.

We obtained 5,348 bp of the genomic DNA sequence across the *PnDREB68* and *PnDREB69* loci through four rounds of PCR, each of which produced a fraction of the entire sequenced region (Figure 1A). This genomic region was re-sequenced in 65 unrelated individuals (GenBank accession numbers KJ019894–KJ020023). The structure of the amplified region is illustrated in Figure 1A, showing a tail-to-tail orientation for transcription of the two genes, which is consistent with that in *P. trichocarpa*. Within this region, *PnDREB69* is intronless, with a 696-bp CDS and a 996-bp 5′ upstream sequence, while *PnDREB68* possesses one intron (75 bp) that separated the CDS into two fragments (456 bp and 300 bp, respectively), and a 1,395 bp 5′ upstream sequence. Regarding the 5′-upstream sequences, we assigned the region upstream of the partial 5′UTR (obtained from cloned cDNA) as the promoter for *PnDREB69* (903 bp) and *PnDREB68* (1284 bp), as the transcription start sites (TSS) for the two genes were not experimentally determined. A 1,430-bp intergenic sequence between the translation stop codons of the genes was also amplified (Figure 1A). Two overlapping PCR primers (RP2 and FP3) (Table S2) covered a 45-bp sequence in the intergenic region (Figure 1A). We separated the intergenic sequence in the middle of the region comprising the two primers and assigned the upstream and downstream sequences to the *PnDREB69* and *PnDREB68* loci, respectively, to perform molecular genetic analyses for each gene. The cDNA sequences were further confirmed by analyses of their genomic DNA sequences.

A 903-bp promoter sequence of *PnDREB69* and *PnDREB68* were compared to infer whether differences in regulatory elements could contribute to the different expression profiles (see below) between the two genes. Various *cis*-acting elements were detected, in which eleven were found to be shared by *PnDREB69* and *PnDREB68* with differences in copy number in most cases (Table S3). Moreover, heat stress-responsive (HSE), drought-inducible (MBS), and auxin-responsive elements were detected in the promoter region of *PnDREB68* but not in *PnDREB69*, while a salicylic acid-responsive (TCA) element was only observed in the *PnDREB69* promoter. Alignment of amino acid sequences revealed conserved DREB1 signature sequences shared by both proteins, including a nuclear localization signal (NLS), an AP2 DNA-binding domain, and a DSAW motif (Figure 1B). A LWSY motif was also identified in PnDREB68, but it was modified to LWSS in PnDREB69. A putative C-terminus activation domain similar to *Arabidopsis* AtDREB1B/CBF1 could also be observed for PnDREB69 and PnDREB68 (Figure 1B).

### Expression Patterns of *PnDREB68* and *PnDREB69* under Normal Growth Conditions and under Abiotic Stress

**Figure 2:**
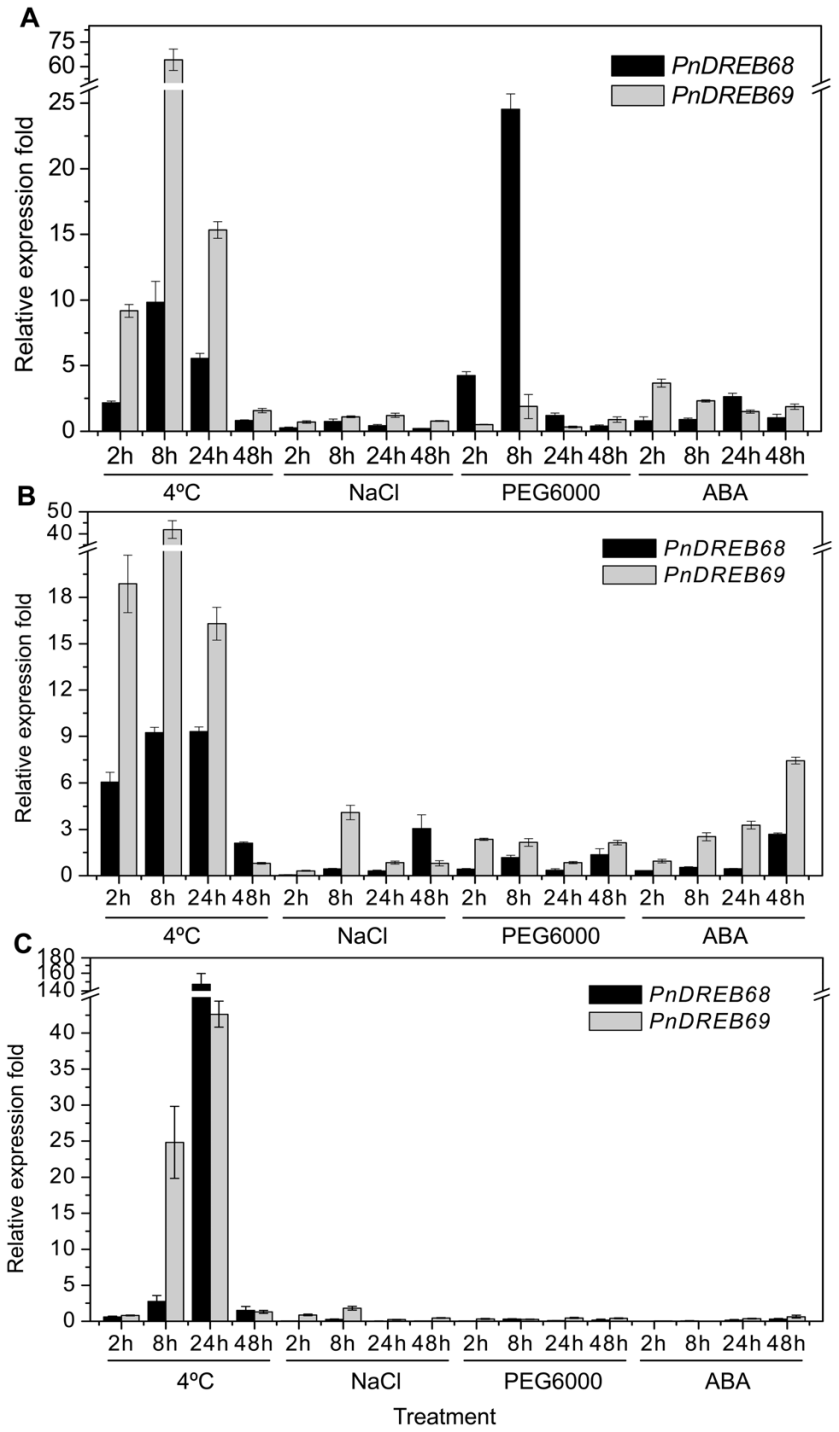
Quantitative RT-PCR analysis of expression patterns of *PnDREB69* and *PnDREB68* in response to abiotic stresses and ABA treatment in (A) leaf, (B) stem, and (C) root tissues. The expression levels for each gene after treatment were normalized to the expression level under normal growth conditions, which was set at 1.0. The relative expression levels of the genes were calculated using the standard 2^−ΔΔCt^ method.

The expression patterns of the duplicated *DREB68* and *DREB69* genes were determined by quantitative RT-PCR under normal growth conditions, abiotic stress conditions, and ABA treatment. The expression of *PnDREB68* and *PnDREB69* was rapidly induced by cold stress in leaf and stem tissues (more than two-fold higher than control levels at the 2 h time point), and a slow response to cold stress was observed in roots (with higher relative abundance than the control beginning at the 8 h time point) (Figure 2A, 2B. and 2C). In most cases, *PnDREB69* had higher expression levels than *PnDREB68* when the plants were exposed to cold stress. When salt stress (NaCl) was applied, *PnDREB69* and *PnDREB68* displayed elevated expression levels in stems at the 8 and 48 h time point, respectively (Figure 2B), but the expression levels were reduced in roots (Figure 2C). Interestingly, in leaf tissue, dehydration (PEG6000) stress significantly induced the expression of *PnDREB68* at the early stage of treatment (2 and 8 h) but not the expression of *PnDREB69* (Figure 2A), whereas in stem tissue, both genes were slightly induced (with different relative abundances) by dehydration (Figure 2B). ABA treatment induced the expression of *PnDREB69* at all time points in leaves and stems, while *PnDREB68* showed elevated expression only at a single time point (24 h in leaves and 48 h in stems) (Figure 2A,2B). Very few transcripts were detected in root tissues under dehydration or ABA treatment conditions (Figure 2C), which suggested that the expression levels for both genes were reduced. The microarray analysis of orthologs from *P. trichocarpa* showed that the expression levels of *DREB68* were higher than those of *DREB69* across most samples, with the exception of xylem tissue. In mature leaf and seedlings grown in continuous darkness, the two genes exhibited inverse trend of transcript accumulations (Figure S1).

### Phylogenetic Relationships and Motif Composition

**Figure 3:**
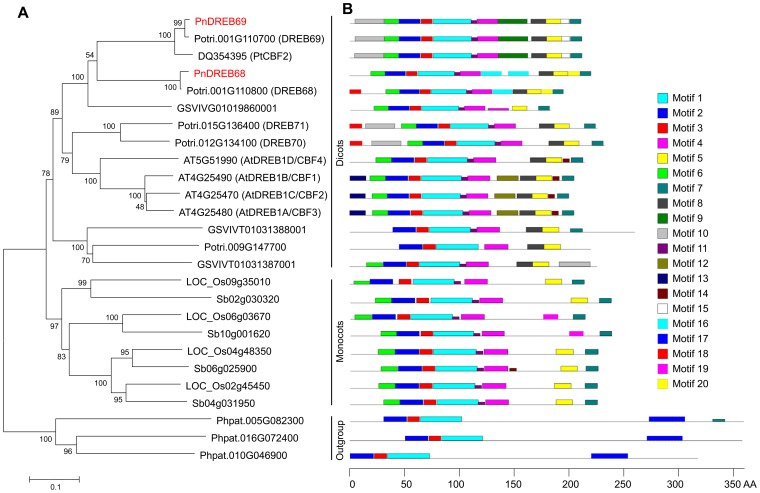
Phylogenetic relationships (A) and conserved motif analysis (B) of DREB1 members from poplar and other plant species. (A) Three DREB2 proteins from *Physcomitrella patens* (Phpat) were used as outgroups. The sequence of PtCBF2 from *P. tomentosa* was obtained from GenBank of the National Center for Biotechnology Information (http://www.ncbi.nlm.hih.gov/). Protein sequences of *P*. *trichocarpa* (Potri), *Arabidopsis thaliana* (AT), *Vitis vinifera* (GSVIV), *Oryza sativa* (LOC_Os), *Sorghum bicolor* (Sb), and *P*. *patens* were downloaded from Phytozome v9.1 (http://www.phytozome.net/). (B) Motifs are illustrated as boxes with different colors.

To investigate the evolutionary relatedness of duplicated *PnDREB68*/*PnDREB69* with the A1 group of DREB (*DREB1*) genes in other plant species, a NJ phylogenetic tree was constructed based on 26 sequences from *P. nigra* and seven additional land plant species. The rooted phylogenetic tree (based on DREB1 proteins) shows that *PnDREB69* and *PnDREB68* have the closest relationships with the homologs in *P. trichocarpa* (*DREB69* and *DREB68*) and *Populus tomentosa* (PtCBF2; Figure 3A). The phylogenetic tree forms two well-defined branches. One branch contains only sequences from dicots, while the other branch contains sequences from monocots, suggesting that the diversification of the *DREB1* genes occurred before the divergence of dicots and monocots.

Twenty distinct motifs were detected in the 26 represented DREB1 proteins using the MEME program (Figure 3B). Details of the 20 motifs were listed in Table S4. Common motif composition occurred in most of the closely related members, which suggested similar functional roles of the motifs among these proteins. Motifs 1, 2, and 3 were most commonly present in all tested proteins. Motif 8 was only found in dicots, while motif 19 was uniquely present in monocots. Additionally, motif 10 appeared to be specific to perennial woody plants (poplar and grape). The basal plant species moss had the lowest number of motifs [47], in which motif 17 was not found in other seed plant species.

### Nucleotide Diversity and Neutrality Tests

Based on the alignment analysis of the 5,348-bp genomic DNA sequences from 64 unrelated individuals, fifty common SNPs (minor allele frequency >0.10) were detected, including three from coding regions and 47 from noncoding regions. Among these SNPs, 11 were identified within *PnDREB69*, while 39 (including three located in CDS) were detected from *PnDREB68* (Figure 1A). Two nonsynonymous SNPs were found in the coding region of *PnDREB68*, causing amino acid changes in the putative activation domain from Val and Ala to Leu and Thr, respectively (Figure 1A). A total of 18 indels were revealed, in which 14, 2, and 2 were located in the *PnDREB68* promoter, *PnDREB69* promoter and intergenic region, respectively (Figure 1A). Of these, eight polymorphic sites (four SNPs and four indels) occurred in several *cis*-acting elements, including the AT1-motif, Box 4, G-box, and TATA-box (Table 1).

**Table 1:** Polymorphisms located in regulatory elements in the promoter regions of *PnDREB69* and *PnDREB68.*

Locus	Polymorphism	Position^a^	Frequency (%)	Regulatoryelement	Functionof element
*PnDREB69*	T/C	−863	35.38	TATA-box	Core promoter element around −30 of transcription start
	A/G	−581	20.00	TATA-box	Core promoter element around −30 of transcription start
	1-base indel	−563	1.54	TATA-box	Core promoter element around −30 of transcription start
*PnDREB68*	1-base indel	−867	18.46	TATA-box	Core promoter element around −30 of transcription start
	C/A	−818	13.85	AT1-motif	Part of a light-responsive module
	1-base indel	−810	3.08	TATA-box	Core promoter element around −30 of transcription start
	A/T	−803	16.92	Box 4	Part of a conserved DNA module involved in light responsiveness
	16-base indel	−462	12.31	G-box	*cis*-Acting regulatory element involved in light responsiveness

a number of base pairs from the translation start site, as the transcription start sites of *PnDREB69* and *PnDREB68* were not experimentally determined.

**Figure 4:**
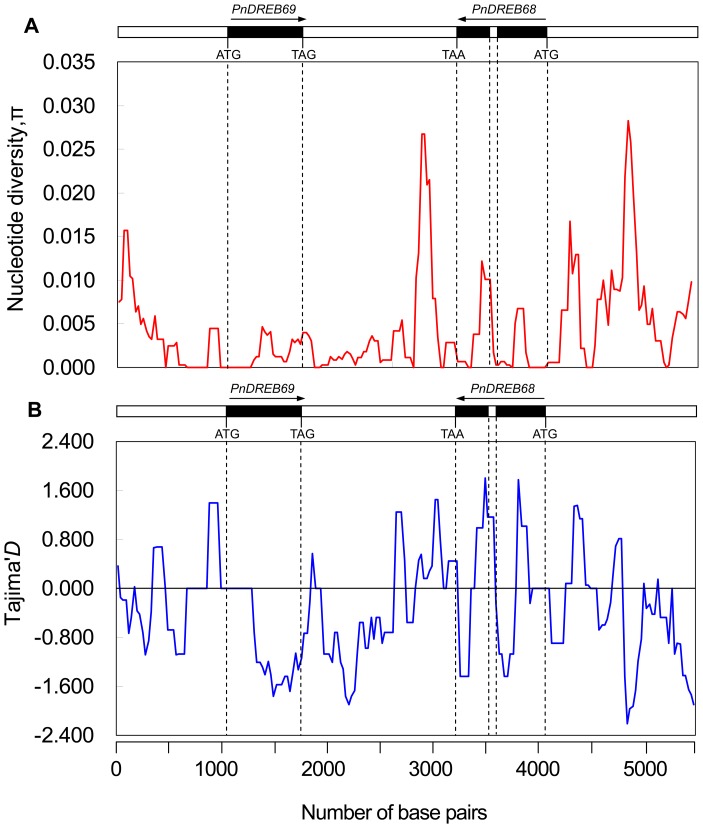
Nucleotide diversity (π) (A) and Tajima’s *D* (B) patterns along the *PnDREB69* and *PnDREB68* loci. The sliding window approach was applied with a step size of 25

Within the entire sequenced region, an analysis of the SNP data found that the promoters and intergenic region showed the highest level of nucleotide diversity relative to coding regions and intron (Figure 1A, Figure 4A). When the two loci were considered separately, a higher average nucleotide diversity was observed for *PnDREB68*, as evidenced by two-fold greater values of θ_W_ (0.00777) and π_T_ (0.00563), than those of *PnDREB69* (θ_W_ = 0.00389, π_T_ = 0.00243) (Figure 4A, Table 2). The overall nucleotide diversity of samples from each geographic region of *PnDREB68* was also found to be higher. Except for the CA samples, larger values of π_sil_, π_syn_, and π_nonsyn_ for *PnDREB68* from the three remaining geographic regions (WE, CE, and SE) were observed, indicating higher levels of nucleotide diversity at these sites. A greater value for the π_nonsyn_/π_syn_ ratio was also inferred for *PnDREB68* relative to that of *PnDREB69* (0.54984 vs. 0.31333). However, compared with *PnDREB68*, samples from CA at *PnDREB69* displayed much higher but similar levels of nucleotide diversity at the syn sites (0.00998 vs. 0.00183) and nonsyn sites (0.00195 vs. 0.00192), respectively. Tests of neutrality of the two genes produced generally negative values for Tajima’s *D* and Fu and Li’s *D*, except for CA at *PnDREB69* and SE at *PnDREB68* for Tajima’s *D* and CA at *PnDREB68* for Fu and Li’s *D*. Within the *PnDREB69* locus, significantly negative Tajima’s *D* values were estimated for the coding region (*D* = −1.85645, *P*<0.05) and nonsyn sites (*D* = −1.96094, *P*<0.05), suggesting that positive selection acted on these sites.

**Table 2:** Nucleotide diversity and neutrality tests in *PnDREB69* and *PnDREB68* from *P. nigra.*

		Polymorphism									
Locus	Geographic regions	*N*	*S*	θ_W_	π_T_	π_sil_	π_syn_	π_nonsyn_	π_nonsyn_/π_syn_	Tajima’s *D*	Fu and Li’s *D*
*PnDREB69*	WE	15	24	0.00309	0.00230	0.00398	0.00171	0.00096	0.56140	−1.19260	−1.69321
	CE	20	17	0.00201	0.00160	0.00190	0.00000	0.00056	0.00000	−0.76189	−0.90690
	SE	18	22	0.00268	0.00226	0.00286	0.00135	0.00021	0.15556	−0.60492	−0.99244
	CA	12	22	0.00305	0.00336	0.00377	0.00998	0.00195	0.19539	0.44303	0.38302
	Total	65	44	0.00389	0.00243	0.00286	0.00300	0.00094	0.31333	−1.24244	−1.40772
*PnDREB68*	WE	15	70	0.00737	0.00528	0.00620	0.00357	0.00164	0.45938	−1.23222	−1.67901
	CE	20	59	0.00571	0.00523	0.00617	0.00310	0.00149	0.48065	−0.34058	−0.14845
	SE	18	57	0.00569	0.00616	0.00733	0.00299	0.00153	0.51171	0.26964	0.15085
	CA	12	39	0.00439	0.00417	0.00473	0.00183	0.00192	0.49180	−0.23144	0.01017
	Total	65	107	0.00777	0.00563	0.00661	0.00311	0.00171	0.54984	−0.95162	−1.96457

WE, Western Europe; SE, Southern Europe; CE, Central Europe; and CA, Central Asia.

### Population Differentiation

We estimated the genetic differentiation within pairs of *P. nigra* populations using the fixation index *F*_ST_. Both genes showed significant genetic differentiation among populations, with a *S*_nn_ value of 0.36564 (*P* = 0.0150) and 0.38653 (*P* = 0.0030) for *PnDREB68* and *PnDREB69*, respectively. No significant pairwise estimate of *F*_ST_ was detected between populations. The results show that low to moderate values of *F*_ST_ were detected for six different population pairs (Western-Southern, Western-Central, Western-Central Asia, Southern-Central, Southern-Central Asia, and Central-Central Asia), ranging from −0.01400 and 0.01953 to 0.17093 and 0.13471 for *PnDREB68* and *PnDREB69*, respectively. Higher *F*_ST_ values were detected when the samples from Central Asia was compared with each of the three geographic regions from Europe (Table 3). A comparison of Central Europe and Central Asia populations yielded the highest value of *F*_ST_ (0.17093) for *PnDREB68*, and a Southern Europe-Central Asia comparison yielded the highest value of *F*_ST_ (0.13471) for *PnDREB69*. A comparison between genes revealed a higher level of genetic differentiation in *PnDREB69* (*F*_ST_ value 0.09189) than in *PnDREB68* (*F*_ST_ value 0.07743).

**Table 3:** Genetic population differentiation of *P. nigra* from four geographic regions.

	*PnDREB68*	*PnDREB69*
Geographic regions	*F*_ST_	*F*_ST_
WE-SE	−0.00275	0.07663
WE-CE	−0.01400	0.03739
WE-CA	0.13004	0.12584
SE-CE	−0.00952	0.01953
SE-CA	0.16574	0.13471
CE-CA	0.17093	0.10741
Total	0.07743*	0.09189**

WE, Western Europe; SE, Southern Europe; CE, Central Europe; and CA, Central Asia. *P<0.05, **P<0.01.

### Linkage Disequilibrium

**Figure 5:**
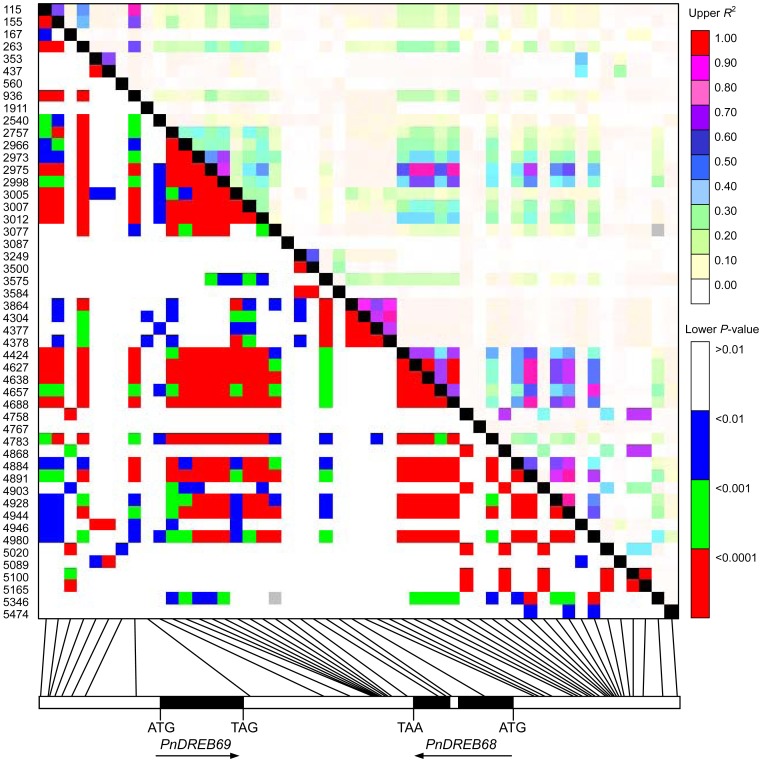
Pairwise linkage disequilibrium (LD) in sequenced region across the *PnDREB69* and *PnDREB68* loci. LD was estimated using the squared allelic correlation coefficient (*r* ^2^) between each pair of SNPs. Boxes below represent the genomic organization of the sequenced region as described in Figure 3. The physical position of each common SNP is labeled on the left and is linked to the corresponding location on the schematic diagram of the genomic region. Each grid in the upper-right matrix represents the level of LD reflected by *r* ^2^ for comparison of each SNP pair.

The extent of linkage disequilibrium (LD) across the *PnDREB69* and *PnDREB68* loci, including the coding regions, intron, intergenic region, and promoters, were estimated using *r* ^2^ as the parameter. A total of 50 common SNPs (frequency >0.10) were included in this analysis, yielding 1,225 pairs of sites for the *r* ^2^ calculations. The average level of LD was low, with an *r* ^2^ value of 0.140251 for the entire region sequenced. In addition, *r* ^2^ values of 0.10641 and 0.21352 were estimated for the *PnDREB69* and *PnDREB68* loci, respectively. The architecture of LD is illustrated in Figure 5. The LD values varied across different regions, with higher values for 5′UTR (average *r* ^2^ = 0.3031) and intergenic regions and lower values for exons (average *r* ^2^ = 0.0647) and introns (average *r* ^2^ = 0.0854). Four (from SNP site 3864 to 4378) and five (from SNP site 4424 to 4688) SNPs in high LD with each other were found to form two distinct haplotype blocks (Figure 5). Within each haplotype block, LD among the SNPs was high (*r* ^2^>0.8279).

### Identification of Candidate SNPs Affecting the Carbon Isotope Ratio in *P. nigra*

**Figure 6:**
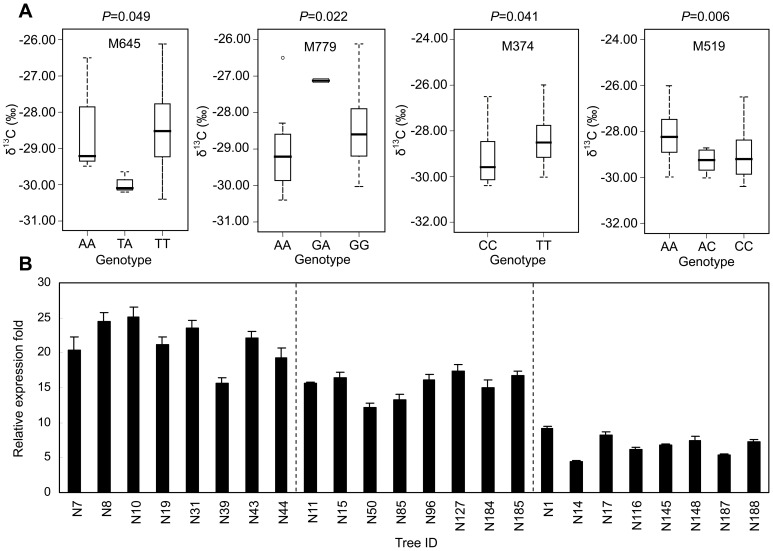
Effects of four SNPs (box plots) of *PnDREB68* on carbon isotope ratios (δ^13^C) (A) and *PnDREB68* expression of three genotype classes of M519 (B) in *P. nigra*. (A) Values of δ^13^C were categorized by genotype class for each SNP. *N = *65 for each SNP examined. (B) For qRT-PCR analysis, 24 representative individuals (8 for each of the three genotype classes) were selected from 65 trees used for SNP identification and δ^13^C measurement. N7–N44 represent the AC group, N11–N185 represent the CC group, and N1–N188 represent the AA group. Expression levels among different genotype classes of M645, M779, or M374, were not significantly different (data not shown).

The expression of *PnDREB68* was strongly induced by dehydration (PEG6000) stress in leaves, which was not observed for *PnDREB69*. PEG6000 treatment is frequently used to mimic drought stress [48]. Therefore, we further measured an important indicator for plant drought tolerance/water use efficiency (WUE), the carbon isotope ratio (δ^13^C), to investigate the natural variation of this phenotype and its potential relationship with SNP polymorphisms. The carbon isotope ratio showed abundant phenotypic variation; the values ranged from −25.99 to −30.40‰ (mean value of −28.49‰). ANOVA indicated a significant difference (*P*<0.05) in δ^13^C values among the 65 individuals tested. This result suggests that the carbon isotope ratio trait bears significant natural variation in *P. nigra* populations and thus is suitable for population genetic analysis. We recorded the genotype (homozygous or heterozygous for each SNP) of each individual tree in our experimental panel (65 individuals), and the carbon isotope ratio of each individual was assigned to each genotyped SNP. For each SNP assayed, phenotypic data from trees with the same genotype were pooled and compared with each other. Significant differences in carbon isotope ratios were found among individuals for four SNPs (M645, M779, M374, and M519) (Figure 6A). M645 and M519 showed three genotype classes whereas M779 and M374 displayed only two genotype classes. All of these SNPs are located in promoter region of *PnDREB68*. No correlation was found between carbon isotope ratios and the SNPs located in *PnDREB69*.

To further examine whether *PnDREB68* expression in the different genotype classes of SNPs is correlated with carbon isotope ratio, expression levels of the four SNPs, M645, M779, M374, and M519, were compared using qRT-PCR. In this analysis, leaf tissues from 24 (8 for each of the three genotype classes of M645, M779 or M519, Figure 6A) or 16 (8 for each of the two genotype classes of M374, Figure 6A) individuals treated with PEG6000 for eight hours were used. These individuals were randomly selected from the 65 trees used for SNP identification and δ^13^C measurement (Table S1). Among these SNPs examined, significant differences (*P*<0.01, ANOVA) in the expression levels among the genotype classes were found only in M519 (Figure 6B). Individuals of AC group showed the highest *PnDREB68* transcript levels, and this gene was expressed at the lowest level in the AA group. These data were negatively correlated with the δ^13^C values observed for the genotype classes of M519 (Figure 6A). In these genotype classes, lower (AC group) and greater (AA group) δ^13^C corresponded with higher and lower expression of *PnDREB68*, respectively.

## Discussion

Gene duplication is believed to be a source of evolutionary forces leading to functional diversification [49], [50]. The consequences of retaining duplicated genes can include the following: (1) retaining the function of its ancestors; (2) loss of gene function (pseudogenization); (3) retaining part of the function of its ancestors (subfunctionalization), and (4) acquiring a new function (neofunctionalization) [51]. Tandem duplication refers to the occurrence of two sequences, one following the other, in the same chromosomal segment. The most remarkable difference in expression patterns revealed in this study is that the expression of *PnDREB68* in leaves was dramatically triggered at the early stage of PEG6000 treatment, whereas no significant increase in expression of *PnDREB69* was observed under the same stress conditions. Expression of *PnDREB69* in leaves and stems was more sensitive to ABA than that of *PnDREB68*, as evidenced by higher expression of *PnDREB69* relative to that of *PnDREB68* throughout the duration of treatment. In leaf tissues, *PnDREB68* may trigger a series of dehydration/drought responses by regulating the expression of downstream genes, while the ABA signal may be essential for the expression and function of *PnDREB69* in both leaves and stems. This phenomenon may suggest functional divergence between the tandemly duplicated *PnDREB68* and *PnDREB69* genes involving in subfunctionalization and/or neofunctionalization, which need to be experimentally validated. The differences in the type and copy number of regulatory elements between the two genes suggested that these differences could be partly responsible for the differential expression of *PnDREB69* and *PnDREB68*. Specifically, the *PnDREB68* promoter possesses a drought-inducible (MBS) element that is absent in *PnDREB69*, which was consistent with the observation that the expression of *PnDREB68* was strongly induced by drought stress (PEG6000) in leaf tissues (Figure 2A). Moreover, the finding of sequence differences in the LWSY(S) motif and the putative activation domain also support the potential functional differences between *PnDREB69* and *PnDREB68*. Expression divergence of orthologs from the close relative, *P. trichocarpa*, was also revealed by microarray analysis, as reflected by distinct expression patterns across a range of tissues, organs and treatments.

The expression of *CBF* genes (DREB1 members) has been well-documented in other plant species. In *Populus balsamifera* subsp. *trichocarpa*, four *CBF* genes (*CBF1*–*CBF4*) are cold-inducible, with various levels of induction observed among genes, depending on the tissue (leaf or stem) in which they are expressed [14]. Divergent expression was also observed among three *CBF* genes in wild tomatoes. These genes are all cold-induced, with various transcript accumulation levels observed among accessions, and they are drought-responsive in *Solanum peruvianum*, although *CBF2* is not drought-responsive in *Solanum chilense* [52]. Genome-wide analysis of the expression pattern of *DREB1* genes has also been reported in *A. thaliana* and rice. The Arabidopsis DREB1 family is composed of six gene members. Among these, *CBF1*–*CBF3* are tandem duplicated and are involved in the cold-response pathway [53], while *CBF4* regulates drought adaptation [11], and *DDF1*–*DDF2* function in the salt response and in development [12], [13]. In rice (*Oryza sativa* L.), six out of ten *DREB1* genes are expressed in response to low temperatures (4°C or 12°C), and two of these genes are also induced by drought or salt stress [54]. Previous studies on other plant species and our data from *P. nigra* show that the response to cold stress is a common phenomenon in plant *DREB1* genes, whereas other expression behaviors such as responses to drought, salt, and ABA are less common.

The phylogenic relationships and conserved motif analysis revealed diversification and a general evolutionary history of plant DREB1 proteins. Two distinct branches in the phylogenetic tree indicated that *DREB1* genes have common ancestors before the split of dicots and monocots (Figure 3A). MEME analysis revealed an increasing number of motifs from moss to seed plants, and several motifs are limited to specific species (dicots, monocots or woody plants) (Figure 3B). These data suggests a possible divergence of functions among different clades of plants in which these motifs are involved. Motif 17 in moss is absent in other seed plants (Figure 3B), which is probably because of a motif-loss event during the evolutionary history of DREB1 proteins.

Evaluations of the level of genetic differentiation can provide information about the extent of population structure, which is crucial for further genetic studies such as association mapping. Significant genetic differentiation in the natural populations was found for both loci (Table 3), where *PnDREB69* displayed a higher fixation index (*F*_ST_) value than *PnDREB68*, indicating a greater level of population differentiation in the former. The degree of genetic differentiation observed in this study was somewhat lower than that previously reported for *P. nigra* (overall *F*_ST_ = 0.268 based on AFLP, and 0.081 based on microsatellite analysis) [24], while still confirming the existence of considerable population subdivision in this species. The estimated level of population differentiation in *P. nigra* is comparable to estimates from other close relative poplar species including *Populus tremula* (*F*_ST_ = 0.117 for five loci) [55], *P. balsamifera* (*F*_ST_ range, 0.018–0.256 for three loci) [56], and *P. tomentosa* (*F*_ST_ range, 0.3552–0.12686 for two loci) [57].

Investigating intraspecific variation of nuclear genes (mainly inferred from the level of nucleotide diversity) in natural populations is essential for understanding the evolutionary forces that act on plant genomes. In this study, we evaluated the nucleotide diversity of two tandemly duplicated *DREB1* genes. We detected two-fold higher total nucleotide diversity (π_T_) for *PnDREB68* than for *PnDREB69* (Table 2), indicating that more relaxed evolutionary constraints occur in *PnDREB68* than in *PnDREB69*. This diversity originated from a much higher value of nucleotide diversity of silent sites (π_sil_) for *PnDREB68* than for *PnDREB69*. The higher level of nucleotide diversity of *PnDREB68* may be a reflection of strong purifying selection acting on this gene. The levels of nucleotide diversity for the two *DREB1* genes are lower than previous estimates for nine loci in *P. nigra* [58]. A comparison within the genus *Populus* revealed significantly lower nucleotide diversity in *PnDREB* loci than in two sucrose synthase genes (*PtSUS1*/*PtSUS2*, π_T_ = 0.00924/0.01093) in *P. tomentosa* [57]. For each gene, the nucleotide diversity of *PnDREB69* is similar to that of *P. balsamifera* (π_T_ = 0.0025) [56] but lower than that of glycosyl-phosphatidylinositol-anchored protein gene (*PtCOBL4*) in *P. tomentosa* [59] and *P. tremula* (π_T_ = 0.0042) [60]. However, *PnDREB68* showed comparable levels of diversity relative to *PtCOBL4* and to that in *P. tremula* but was two-fold higher than that in *P. balsamifera*. This interspecific variation in nucleotide diversity within the same genus can be explained by several factors, including differences in sampling procedures, the genomic regions studied, demographic histories, genetic backgrounds, and mutation rates. Variation in nucleotide diversity was also demonstrated in other *DREB1* genes, including seven *CBF* genes in rye (*Secale cereale* L.; π ranging from 0.0015 to 0.0145) [61] and *Arabidopsis thaliana*, in which the π value of promoters for *CBF* genes ranges from 0.00109 to 0.02749, while the nucleotide diversity of transcript units ranges from 0.00264 to 0.00685 [20]. An analysis of orthologs of three *CBF* genes in wild tomatoes indicated that the average nucleotide diversity ranges from 0.021 to 0.079 in *S*. *chilense* and from 0.016 to 0.106 in *S*. *peruvianum* [52].

Tajima’*D* and Fu and Li’s *D* tests are frequently used estimates that determine whether a gene has departed from neutral evolutionary expectations. The generally negative values for Tajima’s *D* indicate an excess of rare variants in the two genes, which may have resulted from a recent population bottleneck (hitchhiking event) or a process such as background selection. Negative and not significant values for Tajima’s *D* and Fu and Li’s *D* were estimated for the entire region, coding sequence, synonymous sites, and nonsynonymous sites of *PnDREB68*, which is consistent with the results of nucleotide diversity analysis, suggesting that purifying selection was the main force driving the evolution of *PnDREB68*. By contrast, a much lower level of nucleotide diversity was detected for *PnDREB69*, with a smaller nonsynonymous/synonymous nucleotide substitutions (π_nonsyn_/π_syn_) ratio than that observed for *PnDREB68*. Furthermore, a significant negative value for Tajima’s *D* for this locus was inferred for the coding region and for nonsynonymous sites of *PnDREB69*, supporting the hypothesis that this gene has undergone positive selection.

The response to dehydration stress in leaf tissue in *PnDREB68* was absent from the expression patterns observed for *PnDREB69*, which may be associated with positive selection acting on *PnDREB69*. The estimates of LD level for *PnDREB68* or *PnDREB69*, and the entire sequenced region spanning the two loci, are consistent with our previously calculated LD values for nine genes in black poplar, revealing low LD levels in this species [58]. Compared with other species in the genus *Populus*, the strength of LD of black poplar is slightly higher than that of *P. tremula* (squared allelic correlation coefficient, *r* ^2^<0.1; ∼200 bp for 77 genes) [60] and the gibberellin 20-oxidase gene (*PtGA20Ox*) in *P. tomentosa* (*r* ^2^ = 0.1 within a 500-bp region) [62] but much lower than that of *P. balsamifera* (*r* ^2^ = 0.52 for 372 gene fragments) [63]. This interspecific variation in LD levels may be partially attributed to the different gene regions considered, and it also indicates differences in recombination history among species within the genus *Populus*. Despite its high nucleotide diversity, *PnDREB68* exhibited more extended LD than *PnDREB69*, indicating that more favorable mutations were fixed during the evolutionary history of *PnDREB68* than that of *PnDREB69*. This distinct spectrum of LD extension also explains the expression divergence of the two duplicated *DREB1* genes.

DREB family genes have recently been incorporated into association studies of stress-tolerance-related traits in crop species. Two and one nonsynonymous SNPs in *ScCbf15* and *ScCbf12,* respectively, were found to be associated with frost tolerance in rye (*Secale cereale* L.) [64]. Lata et al. reported an association between a synonymous SNP in the *DREB2* subgroup gene *SiDREB2* and dehydration tolerance in foxtail millet (*Setaria italica* L.) [65]. However, molecular evolution and/or association studies of *DREB* family genes have not previously been documented in any woody tree species. In this study, preliminary analysis of phenotypic data and SNP genotypes revealed four noncoding SNPs that may have affected the carbon isotope ratio in the genetic materials studied. QRT-PCR analysis revealed significant differences in expression levels among the three genotype classes of M519, supporting the contribution of this SNP to the natural variation of carbon isotope ratio of black poplar. Based on the fact that plants can discriminate against ^13^C during photosynthesis and the isotopic ratio of ^13^C to ^12^C (δ^13^C) varies in C_3_ plants, the important parameter related to drought tolerance, instantaneous water use efficiency (WUE), is found to be positively related to δ^13^C [66], [67]. This theory has been demonstrated in many plant species [68]. Our results suggest the potential importance of regulatory polymorphisms (located in gene promoters) in contributing to phenotypic variation [69]. Moreover, eight polymorphisms led to nucleotide changes in regulatory elements in *PnDREB69* and *PnDREB68*, including the AT1-motif, Box4, G-box, and TATA-box, which are important for light-regulated gene expression. As *cis*-acting elements in promoters are crucial for gene expression and changes in their sequences could influence gene regulation, further studies need to be conducted on these polymorphisms to address their potential effects on gene expression and phenotypic variation. Two nonsynonymous SNPs with unknown functions were also found in the putative activation domain of *PnDREB68*. An activation domain is essential for the activity of a protein, and this has been demonstrated in *Arabidopsis CBF1*, whose C-terminal 98 amino acids activation domain functions in *trans*-activation. The 14 polymorphisms described above, therefore, are candidates for further association studies of important traits in *P. nigra*, such as water use efficiency/drought tolerance.

In summary, we presented expression and molecular evolution analysis of two duplicated *DREB1* genes in black poplar, *P*. *nigra*. We detected expression divergence; the most remarkable difference was that the expression of *PnDREB68* varied in response to dehydration stress, which was not observed in *PnDREB69*. This result may suggest that functional divergence have occurred between the two genes. *PnDREB68* showed much higher nucleotide diversity than *PnDREB69*, suggesting that strong purifying selection has acted on the former gene. Evidence of positive selection on *PnDREB69* was inferred by neutrality tests, which revealed a significantly negative value of Tajima’s *D* for its coding region. The different evolutionary forces acting on *PnDREB69* and *PnDREB68* may explain the expression divergence observed between these genes. Additionally, the two genes also displayed different degrees of population genetic differentiation and linkage disequilibrium. Finally, a set of polymorphisms in *PnDREB68* and *PnDREB69* may be candidates for association analysis of important traits, such as drought tolerance.

## Supporting Information

Figure S1 **Expression of** ***P. trichocarpa DREB69*** **and** ***DREB68*** **genes across a range of tissues, organs, and treatments.** The patterns of relative transcript accumulation of the two genes were determined by microarray analysis. *Red* indicates higher levels and *blue* indicates lower levels of transcript accumulation. Each column represents the average of biological triplicates. ML, mature leaf; YL, young leaf; RT, root; CL, seedlings grown in continuous light; DL, seedlings grown in continuous darkness and then transferred to light for 3 h; CD, seedlings grown in continuous darkness; XY, differentiating xylem; FC, female catkins; and MC, male catkins.
(TIF)

Table S1 **Geographical origin of the 65 individuals used in this study.**
(DOC)

Table S2 **PCR primers used for RT-PCR, qRT-PCR, and genomic DNA amplifications.**
(DOC)

Table S3 **Putative** ***cis*** **-acting elements in the promoter regions of** ***PnDREB69*** **and** ***PnDREB68*** **.**
(DOC)

Table S4 **Motif sequences of DREB1 proteins used in phylogenic analysis.**
(DOC)

Dataset S1 **Amino acid sequences of DREB1 and outgroup proteins used in phylogenic analysis.**
(DOC)
