# Supplementary figures and images for: Expression and Molecular Evolution of Two DREB1 Genes in Black Poplar (Populus nigra)
Source: PLoS One. 2014 Jun 2;9(6):e98334. doi: 10.1371/journal.pone.0098334 (PMC4041773; doi:10.1371/journal.pone.0098334)

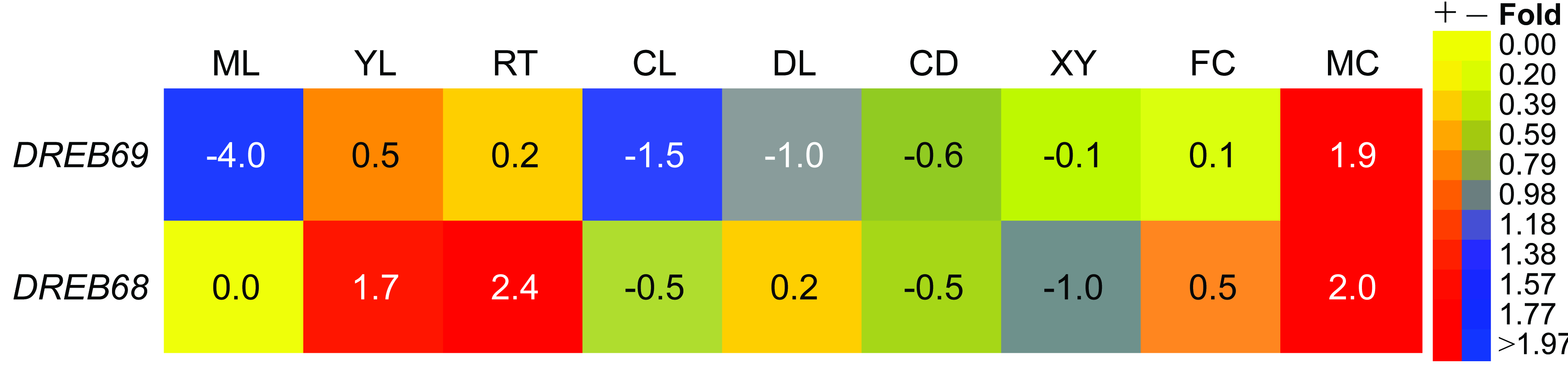

Supplement: Figure S1 — Expression of P. trichocarpa DREB69 and DREB68 genes across a range of tissues, organs, and treatments. The patterns of relative transcript accumulation of the two genes were determined by microarray analysis. Red indicates higher levels and blue indicates lower levels of transcript accumulation. Each column represents the average of biological triplicates. ML, mature leaf; YL, young leaf; RT, root; CL, seedlings grown in continuous light; DL, seedlings grown in continuous darkness and then transferred to light for 3 h; CD, seedlings grown in continuous darkness; XY, differentiating xylem; FC, female catkins; and MC, male catkins. (TIF) [file pone.0098334.s001.tif]
