# Supplementary material for: Expression and Molecular Evolution of Two DREB1 Genes in Black Poplar (Populus nigra)
Source: PLoS One. 2014 Jun 2;9(6):e98334. doi: 10.1371/journal.pone.0098334 (PMC4041773; doi:10.1371/journal.pone.0098334)
Supplement: Table S1 — Geographical origin of the 65 individuals used in this study. (DOC) [file pone.0098334.s002.doc]

**Table S1.** Geographic origin of the 65 individuals used in this study.

| ID | Country | Geographic origin |
| --- | --- | --- |
| E001 | China | CA |
| E002 | China | CA |
| N1 | Belgium | WE |
| N10 | Italy | SE |
| N100 | Hungary | CE |
| N103 | Hungary | CE |
| N11 | Netherlands | WE |
| N110 | Hungary | CE |
| N111 | Hungary | CE |
| N114 | Germany | CE |
| N116 | Germany | CE |
| N117 | Germany | CE |
| N119 | Germany | CE |
| N124 | Germany | CE |
| N126 | Germany | CE |
| N127 | Germany | CE |
| N13 | Slovakia | CE |
| N14 | Slovakia | CE |
| N145 | Netherlands | WE |
| N148 | Netherlands | WE |
| N149 | Netherlands | WE |
| N15 | Serbia | SE |
| N164 | Netherlands | WE |
| N169 | Netherlands | WE |
| N17 | Czech Republic | CE |
| N18 | Czech Republic | CE |
| N181 | Belgium | WE |
| N184 | Belgium | WE |
| N185 | Belgium | WE |
| N186 | Belgium | WE |
| N187 | Belgium | WE |
| N188 | Belgium | WE |
| N189 | Belgium | WE |
| N19 | Rumania | SE |
| N190 | Belgium | WE |
| N20 | Rumania | SE |
| N23 | Germany | CE |
| N25 | Germany | CE |
| N26 | Germany | CE |
| N27 | Germany | CE |
| N29 | Italy | SE |
| N30 | Italy | SE |
| N31 | Italy | SE |
| N32 | Italy | SE |
| N33 | Italy | SE |
| N35 | Italy | SE |
| N36 | Italy | SE |
| N37 | Italy | SE |
| N38 | Italy | SE |
| N39 | Italy | SE |
| N43 | Italy | SE |
| N44 | Italy | SE |
| N45 | Russia | CA |
| N46 | Russia | CA |
| N50 | Russia | CA |
| N51 | Russia | CA |
| N7 | Croatia | SE |
| N8 | Croatia | SE |
| N85 | Russia | CA |
| N96 | Hungary | CE |
| W001 | China | CA |
| W002 | China | CA |
| W005 | China | CA |
| W008 | China | CA |
| W009 | China | CA |

SE, Southern Europe; WE, Western Europe; CE, Central Europe; and CA, Central Asia
