# Supplementary material for: Expression and Molecular Evolution of Two DREB1 Genes in Black Poplar (Populus nigra)
Source: PLoS One. 2014 Jun 2;9(6):e98334. doi: 10.1371/journal.pone.0098334 (PMC4041773; doi:10.1371/journal.pone.0098334)
Supplement: Table S2 — PCR primers used for RT-PCR, qRT-PCR, and genomic DNA amplifications. (DOC) [file pone.0098334.s003.doc]

**Table S2.** PCR primers used for RT-PCR, qRT-PCR, and genomic DNA amplifications

| Gene | Forward primer (5’→3’) | Reverse primer (5’→3’) | Expected product length (bp) | Note |
| --- | --- | --- | --- | --- |
| *PnDREB68* | TCGCCTCATCATTCTATATATTC | TTATGTTTCTTGTCCTTAGTTTCA | 1050 | RT-PCR |
| *PnDREB69* | AATCTCAACACCCTCACAAGT | TTCTGAATTTCTTGAATGTTAAGC | 1020 | RT-PCR |
| *PnDREB68* | ATGTTTGATCGGAGGAATTAATG | ATGCTCTGATGTTTAATAGCCA | 106 | qRT-PCR |
| *PnDREB69* | CTCTCTCCTCCACGTTATGT | GGCACTACGTACTAATCTAGAAG | 115 | qRT-PCR |
| *ACTIN1*^a^ | CATCCAGGCTGTCCTTTCCC | AACGAAGGATGGCGTGTGG | 128 | qRT-PCR |
|  | FP1: CGAATTGAATTGGAAGAAGATTCA | RP1: TCCGCCCTGCACGCTTTTTC | 1242 | Genomic DNA^b^ |
|  | FP2: AGCAGGACTCGCCCAGTTCGAA | RP2: TTTTATGTATGAATGCAATCTT | 1333 | Genomic DNA^c^ |
|  | FR3: CAGGACTTCAGTTTATACAGGAC | RP3: CCAGGGTTGCTCGTGGACATGG | 869 | Genomic DNA^d^ |
|  | FR4: ACGTGGAGGAGAGAGTAGAAGACC | RP4: TGGGTGTCCGGACTAGTTTACACG | 1509 | Genomic DNA^e^ |

^a^ Reference gene

^b^ Primer pair was used to amplify the promoter region and a portion of exon of *PnDREB69*

^c^ Primer pair was used to amplify the upstream sequence of intergenic region and a portion of exon of *PnDREB69*

^d^ Primer pair was used to amplify the downstream sequence of intergenic region and a portion of exon 2 of *PnDREB68*

^e^ Primer pair was used to amplify a portion of exon 2, intron, exon1, and promoter region of *PnDREB68*
