# Supplementary material for: Expression and Molecular Evolution of Two DREB1 Genes in Black Poplar (Populus nigra)
Source: PLoS One. 2014 Jun 2;9(6):e98334. doi: 10.1371/journal.pone.0098334 (PMC4041773; doi:10.1371/journal.pone.0098334)
Supplement: Table S3 — Putative cis -acting elements in the promoter regions of PnDREB69 and PnDREB68 . (DOC) [file pone.0098334.s004.doc]

**Table S3.** Putative *cis*-acting elements in the promoter regions of *PnDREB69* and *PnDREB68*

|  |  | Copy number of elements | |  |  |
| --- | --- | --- | --- | --- | --- |
| Type | Element | *PnDREB69* | *PnDREB68* | Sequence (5’-3’) | Function |
| Light | ACE | 1 | 0 | AAAACGTTTA | *cis*-acting element involved in light responsiveness |
|  | Box 4 | 2 | 4 | ATTAAT | Part of a conserved DNA module involved in light responsiveness |
|  | G-box | 3 | 3 | CACGTG | *cis*-Acting regulatory element involved in light responsiveness |
|  |  | 0 | 1 | tcCACGTGTCACT |  |
|  |  | 1 | 0 | CACGTA |  |
|  |  | 1 | 0 | TAACACGTAG |  |
|  |  | 1 | 0 | GACACGTAGT |  |
|  | GT1-motif | 1 | 0 | GGTTAA | Light-responsive element |
|  | I-box | 1 | 0 | GATAAGATA | Part of a light-responsive element |
|  | AT1-motif | 0 | 1 | ATTAATTTTACA | Part of a light-responsive module |
|  | GATA-box | 3 | 3 | GATA | Required for high level, light regulated, and tissue specific expression |
| Phytohormone | ABRE | 3 | 2 | CACGTG | *cis*-acting element involved in the abscisic acid responsiveness |
|  |  | 1 | 0 | GGACACGTGGC |  |
|  | P-box | 1 | 0 | CCTTTTG | Gibberellin-responsive element |
|  | TCA-element | 1 | 0 | CCATCTTTTT | *cis*-acting element involved in salicylic acid responsiveness |
|  | TGACG-motif | 1 | 0 | TGACG | *cis*-acting regulatory element involved in the MeJA-reponsiveness |
|  | TATC-box | 0 | 1 | TATCCCA | *cis*-acting element involved in gibberellin-responsiveness |
|  | CGTCA-motif | 0 | 1 | CGTCA | *cis*-acting regulatory element involved in the MeJA-responsiveness |
|  | TGA-element | 0 | 1 | AACGAC | Auxin-responsive element |
| Biotic stress | HSE | 0 | 1 | AGAAAATTCG | *cis*-acting element involved in heat stress responsiveness |
|  | MBS | 0 | 2 | (T/C)AACTG | MYB binding site involved in drought inducibility |
| Circadian | Circadian | 1 | 2 | CAANNNNATC | *cis*-acting regulatory element involved in circadian control |
| Basal element | CAAT-box | 6 | 9 | CAAT/CAATT/CAAAT/CCAAT | Common *cis*-acting element in promoter and enhancer region |
|  | TATA-box | 25 | 16 | TATA/TAATA/ATATAT/TTTTA | Core promoter element around -30 of transcription start |
| Other | CCAAT-box | 1 | 2 | CAACGG | MYBHv1 binding site |
|  | Skn-1_motif | 3 | 1 | GTCAT | *cis*-acting regulatory element required for  endosperm expression |
|  | W-box | 2 | 2 | TGAC/TTGAC | WRKY binding site |
|  | GTGA-motif | 6 | 3 | GTGA |  |
|  | AAGAA-motif | 0 | 2 | GAAAGAA |  |

ABRE, abscisic acid-responsive element; HSE, heat stress-responsive element; MBS, MYB binding site; CAANNNNATC, where N represents any base.
