# Supplementary material for: Expression and Molecular Evolution of Two DREB1 Genes in Black Poplar (Populus nigra)
Source: PLoS One. 2014 Jun 2;9(6):e98334. doi: 10.1371/journal.pone.0098334 (PMC4041773; doi:10.1371/journal.pone.0098334)
Supplement: Table S4 — Motif sequences of DREB1 proteins used in phylogenic analysis. (DOC) [file pone.0098334.s005.doc]

**Table S4.** Motif sequences of DREB1 proteins used in phylogenetic analysis.

| Motif | Width | Best possible match |
| --- | --- | --- |
| 1 | 38 | KSRIWLGTFPTAEMAARAHDVAALALRGRSACLNFADS |
| 2 | 21 | AGRKKFKETRHPVYRGVRQRN |
| 3 | 11 | GKWVCEVREPN |
| 4 | 21 | PISTCHKDIQRAAAEAAELFQ |
| 5 | 15 | GLYANMAQGMLLPPP |
| 6 | 15 | SDEYVTLATSCPKKR |
| 7 | 11 | EIDCDVSLWSY |
| 8 | 15 | ENVFYMDEEAMFNMP |
| 9 | 29 | TSDLGGQVMEDFRREDRGEVCCSTNDDIR |
| 10 | 29 | SNSFSIDKQECSYSSLLSDSSGSQQDSPC |
| 11 | 6 | AWRLPV |
| 12 | 20 | TTDHGLDMEETLVEAIYTPE |
| 13 | 15 | MNSFFAFSEMFGSDY |
| 14 | 6 | VQWNHN |
| 15 | 8 | HYTGDWND |
| 16 | 21 | QEFGGHPAKQQDSNAEAVFDM |
| 17 | 33 | TMTDISAFSKDLWTELACHGDISETVLESDQSN |
| 18 | 12 | MDIFCHYSDQNP |
| 19 | 13 | YYYDGMGGGGEWQ |
| 20 | 11 | YVRNDCNDLDH |
