# Supplementary material for: Expression and Molecular Evolution of Two DREB1 Genes in Black Poplar (Populus nigra)
Source: PLoS One. 2014 Jun 2;9(6):e98334. doi: 10.1371/journal.pone.0098334 (PMC4041773; doi:10.1371/journal.pone.0098334)
Supplement: Dataset S1 — Amino acid sequences of DREB1 and outgroup proteins used in phylogenic analysis. (DOC) [file pone.0098334.s006.doc]

>PnDREB68

MPNDRQEASSFSDSSTSRRVVHSDEEVLLATSFPKKRAGRRIFRETRHPVFRGVRKRNGNKWVCEMREPNKKSRIWLGTYPTPEMAARAHDVAALALRGKSACLNFADSAWRLPVPVSKDSKDITRAANEAAELFRPQEFGGHPAKQQDSNAEAVFDMPAELFRPQEFGGHLAKQQDSNTVLEDYSSEVCSDDYKTFQENDVFFEEAVFDMPGLLVDMAEGLLLSPPRYVRNDGNDLDHMENGSDFSLWSY

>PnDREB69

MVMGGSNSFSPDKQESSLSSLLSDSSGSQQDSPSSNEKVLLATSRPKKRAGRRIFKETRHPIFRGVRKRNGDKWVCELREPNKKSRIWLGTYPTPEMAARAHDVAALAFRGKSACLNFADSAWRLPAPISNEAKDIRRAASEAAELFRTSDLGGQVMEDFRREDRGEVCCSTNDDIRDLPSENVGYIDEEAEFNMPGLLANMAEGLLLSPPHYTGDWNDGEIDADWSLWSS

>Potri.001G110800 (DREB68)

MDNFCQFSDQHPLRSNSMPNDRQEASSFSDSSTSRRVVHSDEEVLLATSFPKKRAGRRIFRETRHPVFRGVRKRNGNKWVCEMREPNKKSRIWLGTYPTPEMAARAHDVAALALRGKSACLNFADSAWRLPVPVSKDSKDITRAANEAAELFRPQEFGGHPAKQQDSNAEAVFAMPGENDVFFEEAVFDMPGLLVDMAEGLLLSPPRYVRNDCNDLDHMENGSDLSLWSY

>Potri.001G110700 (DREB69)

MVMGGSNSFSPDKQESSLSSLLSDSSGSQQDSPSSNEKVLLATSRPKKRAGRRIFKETRHPIFRGVRKRNGDKWVCELREPNKKSRIWLGTYPTPEMAARAHDVAALAFRGKSACLNFADSAWRLPVPISNEAKDIRRAASEAAELFRTSDLGGQVMEDFRREDRGEVRSSTNDDIRDLPSENVGYIDEEAEFNMPGLLASMAEGLLLSPPHYTGDWNDGEIDADWSLWSS

>Potri.009G147700 (DREB66)

MEFENYSSSQSSIRQTLSCKQCNSPDIYNLPRGQERPVAVLKKNKAGRKKFKETRHPVYRGVRRRNGNKWVCEVREPNKKSRIWVGTFKSPEMAARAHDVAALALKGELAALNFLDSALILPRAKSSSARDIQRAALAATEPFSNMVMSCSNNCCSEKVPNHSNATFFDEEALFNMPGLLDSMAEGLILTPPAMARGVYWDDMACSTDLTLWEDDYLDSQ

>Potri.015G136400 (DREB71)

MDVFCSYSDQNPIGSMSLLSVLDEQECSYSSVLSDSSITSSVTKGVQPGAIFSDEEVILASRNPKKRAGRKKFRETRHPVYRGVRRRNSGKWVCEVREPNKKSRIWLGTFPTADMAARAHDVAALALRGRSACLNFADSAWRLPTPASSDAKDIQKAAAEAAEAFRPEGSLGVELTRTGDEVEKVAGTAAGDVFYMDDDADFGMPGLLANIAEGMLLPPPNCCGYSGGDSLDNMENNDTDMSLWSFSV

>Potri.012G134100 (DREB70)

MDLFSHYSDPSPFGATDFWSVFNENNGINQEQCSYSPVLSDSSISSNVTTRVQPAPNFSDEEVMLASRNPKKRAGRKKFRETRHPVYRGVRRRNSGKWVCEVREPNKKSRIWLGTFPTAEMAARAHDVAALALRGRSACLNFADSAWRLPVPASSEAKDIQKAAAEAAGGFRPEGCVGGELMRTGDEGEKAAETTAEAGEEVFYMDDEAVFGMPGLLANMAEGMLLPPPHCGGGGDGWDNMENIDADMPLWSFSI

>DQ354395 (PtCBF2)

MVLAGSNSFSADKQESSLSSLLSDSSGSQQDSPCSNEKVLLAASRPKKRAGRRIFKETRHPIFRGVRKRNGDRWVCELREPNKKSRIWLGTYPTPEMAARAHDVAALAFRGKSACLNFADSAWRLPAPISNEAKDIRRAAGEAAELFRTSDLGGQVMEDHTTEDRGEVCSSTNDDIRDLPSENVSYIDEEAESNMPWLLANMAEGLLLSPPHYTGDWNDGEIDADWSLWSS

>GSVIVG01019860001

MELKLFDTPESSSASDWQTRRTIQSDEEVLLASDRPKKRAGRRKFKETRHPVYRGVRRRNGNKWVCELREPNKKSRIWLGTYPTAEMAARAHDVAALAFRGRKACLNFADSAWSLPVPVSKDSMEIRRAAAAAAEAFRPQEFQDHSSGDARKESVEAAESLIFDTAGLLITMAEEPLHSPPPCLGDGFMWEDVELDADMSLWSYSI

>GSVIVT01031388001

MDLDRESSASSSSPTSSPSRANPVSSDSRCPSRCIPHKRKTGRKKFRKTRHPIYRGVRQRNENKWVSEVREPSKKSRIWLGTFPTPEMAARAHDAAALALRGHFASLNFPDSAWRLPRARSSSAGDVQFAAIQAAKAFQQPPSSSSSTPFVMDNMSAGEKAGDGFRTAFVDEEAMFNMPGLIDSMAEGLLLTPPAMCEGFSWDDAVSHIDLSLWNHDFLFFLNVWLCHSNPFKEFTLGAFMGSTKFKLEFGILCWALQVETKGLW

>GSVIVT01031387001

MESERDQSSPSSSSSSSQTKCSISSSPVHKRKAGRKKFRETRHPVYRGVRQRNGNRWVCEVRDPKTKSRIWLGTFSTPEMAARAHDVAALAFRGNFAALNFPDSASRLPRAKSSSAGDIQVAALAAAMAFHSPQLESRKKVVGVALEDSESSEGAPYGSSTVFMDEEALFNMPGLINSMAEGLLLAPPTMLGGFIHCLVFTFSLLHQFYFLFPSLSDDSGKDFHCGCNKLNIKI

>AT5G51990 (AtDREB1D/CBF4)

MNPFYSTFPDSFLSISDHRSPVSDSSECSPKLASSCPKKRAGRKKFRETRHPIYRGVRQRNSGKWVCEVREPNKKSRIWLGTFPTVEMAARAHDVAALALRGRSACLNFADSAWRLRIPETTCPKEIQKAASEAAMAFQNETTTEGSKTAAEAEEAAGEGVREGERRAEEQNGGVFYMDDEALLGMPNFFENMAEGMLLPPPEVGWNHNDFDGVGDVSLWSFDE

>AT4G25490 (AtDREB1B/CBF1)

MNSFSAFSEMFGSDYEPQGGDYCPTLATSCPKKPAGRKKFRETRHPIYRGVRQRNSGKWVSEVREPNKKTRIWLGTFQTAEMAARAHDVAALALRGRSACLNFADSAWRLRIPESTCAKDIQKAAAEAALAFQDETCDTTTTNHGLDMEETMVEAIYTPEQSEGAFYMDEETMFGMPTLLDNMAEGMLLPPPSVQWNHNYDGEGDGDVSLWSY

>AT4G25470 (AtDREB1C/CBF2)

MNSFSAFSEMFGSDYESPVSSGGDYSPKLATSCPKKPAGRKKFRETRHPIYRGVRQRNSGKWVCELREPNKKTRIWLGTFQTAEMAARAHDVAAIALRGRSACLNFADSAWRLRIPESTCAKEIQKAAAEAALNFQDEMCHMTTDAHGLDMEETLVEAIYTPEQSQDAFYMDEEAMLGMSSLLDNMAEGMLLPSPSVQWNYNFDVEGDDDVSLWSY

>AT4G25480 (AtDREB1A/CBF3)

MNSFSAFSEMFGSDYESSVSSGGDYIPTLASSCPKKPAGRKKFRETRHPIYRGVRRRNSGKWVCEVREPNKKTRIWLGTFQTAEMAARAHDVAALALRGRSACLNFADSAWRLRIPESTCAKDIQKAAAEAALAFQDEMCDATTDHGFDMEETLVEAIYTAEQSENAFYMHDEAMFEMPSLLANMAEGMLLPLPSVQWNHNHEVDGDDDDVSLWSY

>LOC_Os04g48350

MEWAYYGSGYSSSGTPSPVGGDGDEDSYMTVSSAPPKRRAGRTKFKETRHPVYKGVRSRNPGRWVCEVREPHGKQRIWLGTFETAEMAARAHDVAAMALRGRAACLNFADSPRRLRVPPLGAGHEEIRRAAVEAAELFRPAPGQHNAAAEAAAAVAAQATAASAELFADFPCYPMDGLEFEMQGYLDMAQGMLIEPPPLAGQSTWAEEDYDCEVNLWSY

>LOC_Os02g45450

MDVSAALSSDYSSGTPSPVAADADDGSSAYMTVSSAPPKRRAGRTKFKETRHPVFKGVRRRNPGRWVCEVREPHGKQRIWLGTFETAEMAARAHDVAALALRGRAACLNFADSPRRLRVPPIGASHDDIRRAAAEAAEAFRPPPDESNAATEVAAAASGATNSNAEQFASHPYYEVMDDGLDLGMQGYLDMAQGMLIDPPPMAGDPAVGSGEDDNDGEVQLWSY

>LOC_Os06g03670

MEYYEQEEYATVTSAPPKRPAGRTKFRETRHPVYRGVRRRGPAGRWVCEVREPNKKSRIWLGTFATAEAAARAHDVAALALRGRGACLNFADSARLLRVDPATLATPDDIRRAAIELAESCPHDAAAAAASSSAAAVEASAAAAPAMMMQYQDDMAATPSSYDYAYYGNMDFDQPSYYYDGMGGGGEYQSWQMDGDDDGGAGGYGGGDVTLWSY

>LOC_Os09g35010

MEVEEAAYRTVWSEPPKRPAGRTKFRETRHPVYRGVRRRGGRPGAAGRWVCEVRVPGARGSRLWLGTFATAEAAARAHDAAALALRGRAACLNFADSAWRMPPVPASAALAGARGVRDAVAVAVEAFQRQSAAPSSPAETFANDGDEEEDNKDVLPVAAAEVFDAGAFELDDGFRFGGMDAGSYYASLAQGLLVEPPAAGAWWEDGELAGSDMPLWSY

>Sb04g031950

MDAASFSDYSSGTPSPVGGVSGGVDGDDGGSSSSYMTVSSAPPKRRAGRTKFKETRHPVYKGVRRRNPGRWVCEVREPHGKQRIWLGTFETAEMAARAHDVAALALRGRAACLNFADSPRLLRVPPMGSGHDEIRRAAAVAADQFRPAPDRQGNVATAEEAADTTPLDATTQSVVDDPYCIIDDRLDFGMQGYLDMAQGMLIDPPPMAGSSTSGGDDDDDGEVKLWSY

>Sb06g025900

MDWAYYGISGYSTTPSPPPVGVGGGVGDEDEAYMTVSSAPPKRRAGRTKFKETRHPVYKGVRSRNPGRWVCEVREPHGRQRIWLGTFETAEMAARAHDVAALALRGRAACLNFADSPRRLRVPAQGAGHDEIRRAAVEAAELFRPPQQQQHNVGGSEAAAAAVAAPCAQGSGGGGIGGDFAYYPMDDGLEFEMHGYLDMAQGMLVDPPQATAWIEDEYECEVSLWSY

>Sb10g001620

MEYGVADDYGYGYGGYDDQQDLPSSSSVDGDEYATVLSAPPKRPAGRTKFRETRHPVYRGVRRRGPAGRWVCEVREPNKKSRIWLGTFATAEAAARAHDVAALALRGRAACLNFADSARLLRVDPATLATPDDIRRAAIQLAEDSSSSTPDASAAAAAVAVASSASVGQATPSSSAYQAGDDATGAAMYGAEYAAAAMYGAGMDFDHSYYYDGMVGGNEWQSAGSSGWHSNVDAGDDEGAGDMSLWSYY

>Sb02g030320

MCPPIKKEMSAESSASASASPSASSEHQTVWTSPPKRPAGRTKFRETRHPVFRGVRRRGNAGRWVCEVRVPGRRGCRLWLGTFDTAEAAARAHDAAMLAIAGAGACLNFADSAWLLAVPASYASLAEVRHAVAEAVEDFQRREGEGEAAAGEDDARSATSSSVPSTSSGNEDDAAATDGEESSPATEDSSPFEMDVFNDMSWDLYYASMAQGMLMELPSAVPAFGDDGYANVADVPLWSY

>Phpat.016G072400

MQHGSSVAETLAWWASRNNGIGGKAGANQADDSNGPRKIIRKAPARGSKKGCMKGKGGPENAMCNYRGVRQRTWGKWVAEIREPNRGSRLWLGTYPTAEIAALAYDSAARVLYGSNALLNLPGETASPVAGTASTSATSASSAEIAISDDRSSAKSERVTAKRTPAKHTGKPNNSAASFTDPDPSATLDTATSLSQHQAAEVALSYDLLEPLPRHPKPEPVQELQLSAEPSFPPLLQEPQEPMDCNDLLGKEFDLFDFKMDEGDMQLPPSLKTNSNSSTMTDSSAFSRDLWTELACHGDISETVLDSDHSIASSTTLGEDKPEDVHVLRSLTEDDMQRLTMPESPEVTLFEISPSLNRKEAWTSLLE

>Phpat.010G046900

MKGKGGPENAQCSYRGVRQRTWGKWVAEIREPNRGSRLWLGTYGTAEEAALAYDEAARVLYGLNALLNLPDRGPTAPASNFGVSEGSRNNQENSGLGYRKSNLGGGPTESAASGTVPDGQDEGLDTADSGSPRKLLEASIAEMSRTSPRRLAAEAILPESSSSWLEDNDAHEPNEALDLEKVRNEQIVFNDLLDVEDLDILDPKLAELPRLLRSNSDSSTMTDSSVFSKDLWEELACHMTISDGTLESGKSNDSSSLTANNMEKHDVRCDDVEGICISDGQLVSPYSPEVEMLDNSPLMQKKQAWTTLLSDQASPS

>Phpat.005G082300

MGKTDAGSGSSRSCTKKNMRQAPAKGSKRGCMKGKGGPENALCTYRGVRQQTWGMWVAEIRKPNRGLRLWLGTYSTAEIAALAYNSAARILYGPNALLNQPNRTPNGSNPDEHMDRTTATYSSASSAEIGNLGVCTLSHRGKSFQKIERVPCKLRSGGAEPHSQAGKEGRSAASFTTPESLADHDIDTATSLQALQMLAADVVLPDSEPPKPWPCDPKPEFIDKAWLQESNQPSFLPPKQLIKDVLENDHDIFNFKLDNGSDLAPPLQLETTSGLNEITTTIGAFSEARWTKNECGSDMPEAMPESDQSNGSTFTSTTHTMDTTVDVASLGDLNDEDLQILMQLWSPELASYAWNAASVELSVSR
